# One stage surgical treatment of aortic valve disease and aortic coarctation with aortic bypass grafting through the diaphragm and aortic valve replacement

**DOI:** 10.1186/s13019-015-0338-2

**Published:** 2015-11-10

**Authors:** Zipu Yu, Shengjun Wu, Chengchen Li, Yu Zou, Liang Ma

**Affiliations:** Department of Cardiac Surgery, 1st Affiliated Hospital, Zhejiang University, 79 Qingchun road, Hangzhou, Zhejiang China

**Keywords:** Aortic bypass grafting, Aortic coarctation, Aortic valve disease, One-stage surgical treatment

## Abstract

**Objectives:**

To validate ascending aorta-lower abdominal aorta bypass grafting treatment for patients with descending aortic coarctation and an aortic valve disease.

**Methods:**

The three patients in whom a descending atypical aortic coarctation was associated with an aortic valve disease were treated with one stage surgical treatment with aortic bypass grafting through the diaphragm and aortic valve replacement in our heart center. Operative technique consisted of performing ascending aorta-lower abdominal aorta bypass grafting through diaphragm muscle and implementing aortic valve replacement. The mean time for extracorporeal circulation and occluding clamp of aorta was recorded. Blood pressure data for pre- and post-operation was measured in the limbs. Computer-enhanced transvenous angiograms of pre- and post-operation were applied for detection of aortic stenosis. The other adverse events were noticed in outpatient service during a follow-up period.

**Results:**

The mean extracorporeal circulation time was 54 ± 11 min. The mean time for occluding clamp of aorta was 34 ± 6 min. An arterial pressure gradient was totally corrected after surgical treatment. Post-operation computer-enhanced transvenous angiograms showed the grafts to be open with a fluent flow. The patients had no gastrointestinal tract complications. No adverse event was noticed during a follow-up period in outpatient service.

**Conclusions:**

Treatment of ascending aorta-lower abdominal aorta bypass is advisable for patients with descending aortic coarctation and an aortic valve disease.

## Background

Repair of descending atypical coarctation is not an easy operation for its particular location. What’s the best surgical treatment for patients with descending aortic coarctation combined with aortic valve disease? Here we will introduce our experience for these cases. Our experience aims at attracting doctors’ attention to aortic bypass grafting from the ascending aorta to lower abdominal aorta as a surgical alternative which will allow an adequate correction of abnormal hemodynamics in patients of this kind.

## Methods

### Patients

**Fig. 1:**
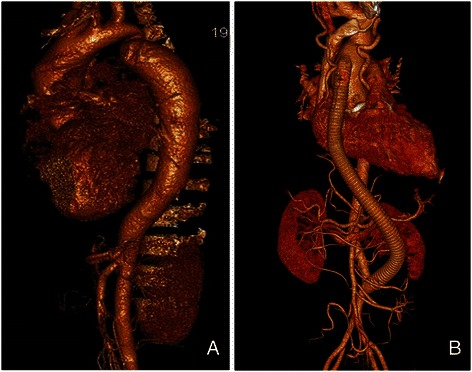
Preoperative thoracic and abdominal computed tomographic angiography (CTA) showing a descending atypical coarctation (**a**). Postoperative thoracic and abdominal CTA showing the excellent fluent flow of the aorta-lower abdomiml aorta bypass graft (**b**)

From March in 2009 to October in 2014, 3 adult men patients were admitted to our center with descending aortic coarctation combined with aortic valve disease at the Department of Cardiovascular and Thoracic Surgery, the First Affiliated Hospital, College of Medicine, Zhejiang University, Hangzhou, China. All patients underwent preoperative assessment including laboratory examination, ultrasonography, thoracic and abdominal computed tomographic angiography (CTA) (Fig. 1). Institutional Review Board approval and informed consent from the patients were obtained to perform one-stage surgical treatment of aortic bypass grafting and aortic valve replacement.

### Operative technique

**Fig. 2:**
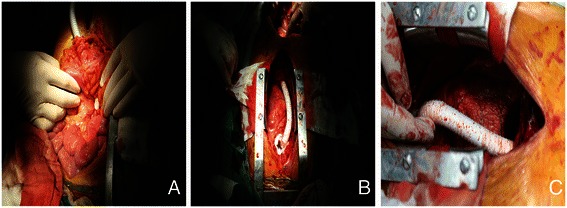
The aorta-lower abdomiml aorta bypass graft procedure. End-to-side graft-to-distal abdominal aorta anastomosis and end-to-side graft-to ascending aorta anastomosis is performed (**a**, **c**). The course of the graft traversed through a hole cut into the diaphragm muscle (**b**)

The patient, under general anesthesia, was placed in the supine position with the head slightly elevated. A midline incision in the sternum was made to expose the heart. Extracorporeal circulation was set up to perform aortic valve replacement. Pathological aortic valve was replaced with Carbomedics mechanical valve. After recover of heartbeating, extracorporeal circulation was withdrawn. A midline incision in the abdomen was made to isolate the abdominal aorta between renal artery and common iliac artery. A crimped woven vascular prosthesis 16 mm in diameter was elected in accordance with diameter magnitude of abdominal aorta. With the aid of a partially occluding clamp, the graft was sniped with a bevel connection and anastomosed end-to-side to the distal abdominal aorta isolated previously. The course of the graft passed the liver anteriorly and transversed mesocolon posteriorly, traversd across the root segment of omentum majus, and then was scheduled to head upward through a hole cut in the diaphragm muscle, passed the heart anteriorly in a gentle curve to get to ascending aorta. Finally it was anastomosed with ascending aorta in the end-to-side manner (Fig. 2). The prosthesis was allowed to be filled with blood and expelled the air trapped in the graft. Then the proximal clamp was removed, the covering of the distal anastomosis with peritoneum and the routine closure of the incision were performed at last. All patients take warfarin all their life.

Caution should be paid to details, such as proper tightness of hole cut in the diaphragm muscle to avoid diaphragmatic hernia, careful handle not to give rise to injury of intra-abdominal organ and appropriate fixation of graft in case of intestinal adhesion.

## Results

The clinical characteristics and outcomes of all patients are summarized in Table 1.

**Table 1:** the clinical characteristics and outcomes of patients

Patient	1	2	3
Age(year)	47	39	48
Follow-up(month)	70	54	5
Outcome	Favorable	Favorable	Favorable
Pre-operation upper-limb blood pressure(mm Hg)	125/65	135/65	145/50
Pre-operation lower-limb blood pressure(mm Hg)	100/55	110/50	125/45
Post-operation upper-limb blood pressure(mm Hg)	125/65	125/80	130/80
Post-operation lower-limb blood pressure(mm Hg)	130/70	130/80	130/75

The mean extracorporeal circulation time was 54 ± 11 min. The mean time for occluding clamp of aorta was 34 ± 6 min. No operative mortality was noted. An arterial pressure gradient in the limbs was totally corrected.

Recent computer-enhanced transvenous angiograms showed the grafts to be open with a fluent flow (Fig. 1). During a follow-up period, no gastrointestinal tract complications were noticed in patients.

## Discussion

Coarctation of the aorta (CoA) is typically a narrowing of the thoracic aorta just distal to the left subclavian artery. The treatment for aortic coarctation has undergone a long time development since the first time Crafoord reported such case. It is a congenital vascular malformation disease, almost accouting for 5–8 % of all congenital heart diseases, which is frequently accompanied with atrial septal defect and ventricular septal defect [1]. Aortic coarctation concomitant with aortic valve disease is scarce, always combined with congenital cardiovascular abnormalities for most cases [2, 3].

Treatment options include surgery, balloon angioplasty and endovascular stenting. Stent implantation has gained great success and become a widely accepted therapeutic option for CoA in children and adults during the past decades [4–12]. However, serious concerns still remain permanently in the interventional treatment of CoA. During the intermediate-term and long-term follow-ups, several studies indicated that the incidence of re-intervention varied between 6 and 20 % after stent implantation [13–19]. Apart from this, the rupture of the aortic wall was commonly observed in old patients with decreased aortic wall compliance and children with a vascular pathology state. Although procedure-related technical complications have decreased, even if rarely, complications may still be observed.

On the contrary, surgical correction is usually recommended for its wonderful results in increasing diameters of coarctation of the aorta and low re-intervention rate [20]. As reported by Brown et al. [21], surgical repair of coarctation produces lasting results in the majority of the patients and remains the gold standard treatment for CoA. Currently, no consensus has been reached for the best treatment of complex coarctation. Coarctation in association with other cardiac pathology can be treated with a one-stage or two-stage approach. Someone puts forward the viewpoint that it should be treated for stages, aortic valve disease treatment followed by aortic coarctation treatment about 2 months later [22]. It is inclined to adopt the one-stage surgical treatment for these cases with the development of surgical techniques and extracorporeal circulation. Classic surgical operations for this include anatomic repair or extraanatomic bypass grafting. The former operation needs completely isolation of aorta, which may bring out massive haemorrhage. The later operation has chances of pseudoaneurysm. As far as the recurrence of coarctation is concerned, there is no statistic differences between them [23]. The latter surgical procedure is more fit for adults, in whom stent implantation has not so excellent effectiveness. Several open techniques of CoA repair have been described, which included extra-anatomic bypass, resection with end-to-end anastomosis (REE) and resection and interposition graft (RIPG). Bouchart et al. described 35 patients, in whom most were treated with REE [24]. Duara et al. had 46 open repair cases including 27 REE and 13 RIPG, both of which produced a favourable therapeutic effect [25]. Roselli compared 60 endovascular repairs with 40 open repairs, which indicated that open techniques of CoA repair was associated low risk [26]. Other studies also reported low perioperative major morbidity and no repair-related mortality with open surgical techniques [27, 28]. However, conventional anatomic repair may be complicated by the need for extensive mobilization of the aorta, control of blood vessels, the possibility of parenchymal lung injury, damage to the recurrent laryngeal or phrenic nerves, the chances of chylothorax and spinal cord ischemia. The most feared complication of aortic surgery is paraplegia and risk of spinal cord injury, which increases with prolonged aortic cross-clamp time and patient age [29].

One-stage surgical treatment of extraanatomic bypass grafting could increase effectiveness and bring down cost, risk and suffering for patients. Cooley and Norman adopt the use of a bypass graft between the ascending aorta and the subdiaphragmatic portion of the upper abdominal aorta for aortic coarctation in children in 1975. Use of this operation skills was also reported by Siderys H and Levy Praschker BG [30, 31]. In order to avoid the risk of the anatomic repair operations, our preferred technique in adults is ascending-to-infrarenal abdominal aorta by-pass grafting, which will not block the blood flow for kidney during the opration. As a result, this treatment reduce the chances of injuries for kidney. In our center during past decades, we have been using ascending-to-infrarenal abdominal aorta by-pass grafting mainly under circumstances for descending aortic coarctation combined with heart valve disease. Advantages for this operation include: (1) completely exposure of surgical field, making anastomosis and hemostasis easier in comparison with ascending-to-descending aortic bypass. (2) not movement of heart to keep stabilities of hemodynamics. (3) avoiding hazard of spinal cord ischemia due to aortic cross-clamping. (4) The prosthesis is placed with a gentle curve, thereby avoiding graft obstruction. (5) The distal anastomosis is embedded in a retroperitoneal position and covered with peritoneum, which decreases the risk of fistula formation and other complications in patients. Disadvantages include: (1) two operation incisions. (2) gastrointestinal function change and intestinal adhesion or obstruction if improper treatment, which were not found in our cases.

The long-term results for our patients are satisfactory. We did not document adhesions and compressions in any patient which points to the safety of the ascending-to-infrarenal abdominal aorta by-pass grafting.

## Conclusions

In conclusion, one-stage surgical treatment of descending aortic coarctation combined with aortic valve disease represents a safer solution. Knowledge of this method should contribute to the treatment of complex congenital anomalies.
